# Several microRNAs could predict survival in patients with hepatitis B-related liver cancer

**DOI:** 10.1038/srep45195

**Published:** 2017-03-21

**Authors:** Ye Zhen, Zhao Xinghui, Wu Chao, Zhao Yi, Chen Jinwen, Gao Ruifang, Zhang Chao, Zhao Min, Guo Chunlei, Fang Yan, Du Lingfang, Shen Long, Shen Wenzhi, Luo Xiaohe, Xiang Rong

**Affiliations:** 1 Department of Tumor Molecular Biology, Nankai University School of Medicine, Tianjin 371000, China; 2 School of public health, Taishan Medical University, Tai’an 271016, China; 3 Department of Infectious Disease, Tai’an Central Hospital, Tai’an 271000, China; 4 Laboratory of Vaccine and Antibody Engineering, Beijing Institute of Biotechnology, Beijing 100071, China; 5 Key Laboratory of Intelligent Information Processing, Institute of Computing Technology, Chinese Academy of Sciences, Beijing, 100190, China; 6 College of Information Science and Engineering, Shandong Agriculture University, Tai’an 271000, China; 7 The 2011 Project Collaborative Innovation Center for Biological Therapy, Nankai University School of Medicine, Tianjin 371000, China; 8 The International Collaborative Laboratory for Biological Medicine of the Ministry of Education, Nankai University School of Medicine, Tianjin 371000, China.

## Abstract

MicroRNAs as biomarkers play an important role in the tumorigenesis process, including hepatocellular carcinomas (HCCs). In this paper, we used The Cancer Genome Atlas (TCGA) database to mine hepatitis B-related liver cancer microRNAs that could predict survival in patients with hepatitis B-related liver cancer. There were 93 cases of HBV-HCC and 49 cases of adjacent normal controls included in the study. Kaplan–Meier survival analysis of a liver cancer group versus a normal control group of differentially expressed genes identified eight genes with statistical significance. Compared with the normal liver cell line, hepatocellular carcinoma cell lines had high expression of 8 microRNAs, albeit at different levels. A Cox proportional hazards regression model for multivariate analysis showed that four genes had a significant difference. We established classification models to distinguish short survival time and long survival time of liver cancers. Eight genes (mir9-3, mir10b, mir31, mir519c, mir522, mir3660, mir4784, and mir6883) were identified could predict survival in patients with HBV-HCC. There was a significant correlation between mir10b and mir31 and clinical stages (p < 0.05). A random forests model effectively estimated patient survival times.

Hepatocellular carcinoma has the third highest malignant tumor mortality worldwide. The appearance of clinical symptoms often indicates the progression of the disease, after which the potential treatment options are very limited. Thus, early identification of tumors is essential to perform potential therapeutic interventions^1^. The main risk factors for liver cancer include hepatitis B (HBV) infection, hepatitis C (HCV) infection, and lifestyle characteristics such as chronic alcohol abuse, nonalcoholic fatty liver disease, diabetes, and obesity^2^,^3^,^4^. Currently, the most widely used biological marker of liver cancer is alpha fetoprotein (AFP), especially in developing countries. AFP does not have good reliability in clinical applications because of limited diagnostic performance and ability. The American Association for the Study of Liver Diseases concluded that AFP lacks sufficient sensitivity and specificity to effectively monitor or diagnose HCC^5^. Because it is initially asymptomatic, HCC has usually progressed to a late stage by the time of diagnosis. Therefore, early detection and a molecular target for a therapeutic liver cancer biomarker are urgently needed^6^.

MicroRNAs are endogenous, non-coding RNAs that regulate gene expression at the post-transcriptional level, and they participate in a variety of biological pathways^7^. MicroRNAs have potential as biomarkers because they can indicate functions that play important roles in tumorigenesis: Studies have found that microRNAs are involved in viral replication and delay and epigenetic modulation and interact with viruses or indirectly activate important cancer related pathways^8^. They also play an important role in normal biological processes and are associated with many tumors, including HCC^9^.

Many studies on the liver tissue, plasma, or polymorphisms have found a correlation between microRNAs and liver cancer. Shi *et al*. found that miR-22 levels were significantly lower in hepatitis B-related HCC (HBV-HCC). Overexpression of miR-22 may inhibit the growth of cancer cells^10^. Compared with hepatitis B cirrhosis, chronic hepatitis B, and healthy controls, serum miR-101 was significantly lower in HBV-HCC^11^. In China, the miR-146a G>C and miR-196a2 C>T polymorphisms were found to be associated with HCC risk, especially in patients with HBV infection. MicroRNA SNP sequences can be used as biomarkers for the diagnosis of liver cancer^12^.

Many of the previous studies on the use of microRNAs as markers for liver cancer used qRT-PCR and gene chip methods to detect microRNAs^13^,^14^. Jian Zhang *et al*. used high-throughput microRNA sequencing data and clinical data from the TCGA (The Cancer Genome Atlas) database (http://cancergenome.nih.gov/) to screen out seven microRNAs that could predict liver cancer prognosis^15^. Shi *et al*. used Gene Expression Omnibus to search for HCC miRNA expression profiling^16^. We believe that there are multiple liver cancer pathogenic factors, for example, HBV, HCV, and alcohol use, and different etiologies could lead to different microRNA expression levels. In this paper, we screened the TCGA database to identify HBV-HCC markers to better understand the relationship between microRNAs and disease progression and prognosis. In addition, we established classification models to predict the prognosis of patients. The results will help to identify approaches for HBV-HCC diagnosis, treatment, and prognosis.

## Results

### Liver cancer group vs. normal control group

**Figure 1:**
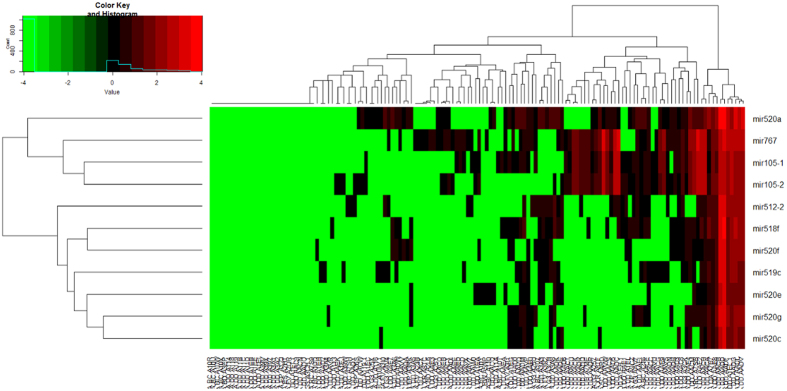
Heat map comparing the liver cancer group with the normal control group.

There were 181 genes upregulated more than three-fold with p < 0.05 (Supplementary Materials, S-1). In addition, there were 18 genes downregulated at least 0.33-fold with p < 0.05 (S-2). The heat map analysis is shown in Fig. 1, and the darker colors represent the higher gene expression levels. The volcano plot shows the distribution of the differentially expressed genes in S-3.

### Survival analysis

**Figure 2:**
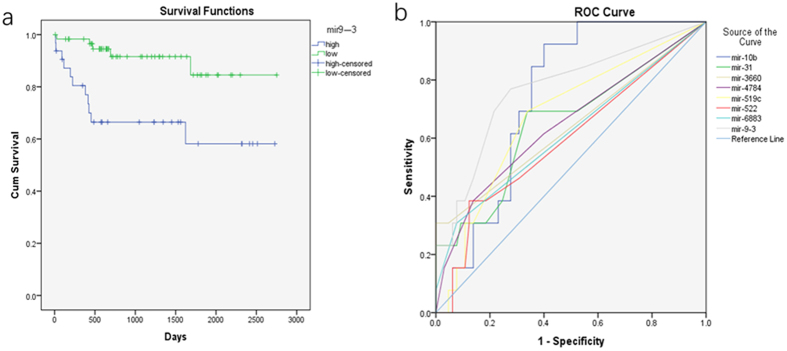
(**a**) Kaplan-Meier survival analysis indicating that low expression of mir9-3 is superior to high expression, p < 0.05. (**b**) ROC curve of eight genes for use in the distinguish G3 and G4 of liver cancer patients.

The Kaplan–Meier survival analysis identified eight genes with statistical significance at p < 0.05 among the differential expression (DE) genes: mir9-3, mir10b, mir31, mir519c, mir522, mir3660, mir4784, and mir6883 (Fig. 2a, S1–4~10). This suggested that low expression of these genes indicated a better survival prognosis than high expression. The Cox proportional hazards regression model for multivariate analysis showed that four genes (mir10b, mir519c, mir3660, and mir6883) were statistically different (Table 1).

### ROC analysis

Mir9-3, mir10b, mir31, mir519c, mir522, mir3660, mir4784, and mir6883 were included in the ROC analysis (There were 13 patients with short survival times for liver cancer (G3) and 65 patients (G4) with long survival times. A short survival time for liver cancer was defined as a total survival period of less than 500 days; a long survival time was more than 500 days. Fig. 2b). Three genes had statistical difference (Mir9-3, mir10b and mir519c). The areas under the curve (AUC) for mir9-3, mir10b, and mir519c were 0.769, 0.738 and 0.676, respectively, with p < 0.05. This suggested that the expression levels of mir9-3, mir10b, and mir519c could effectively distinguish between G3 and G4 liver cancers.

### Principal component analysis

The scree plot and cumulative contribution plot are shown in in S-11. The cumulative contribution rate was 0.857 for the first five genes.

### qPCR validation

**Figure 3:**
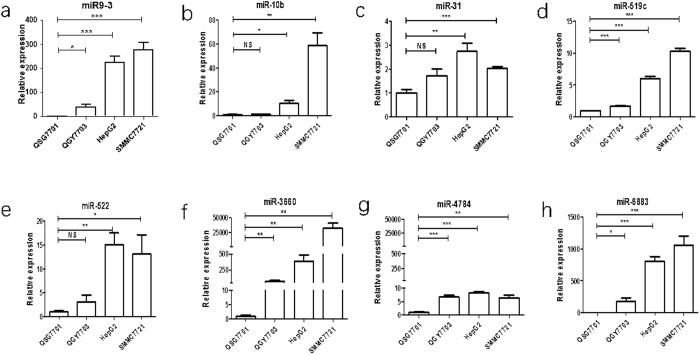
The relative expression levels of 8 microRNAs in normal cell lines compared with liver cancer cell lines.

Compared with the normal liver cell line, hepatocellular carcinoma cell lines had high expression of 8 microRNAs, albeit at different levels. The expression of mi10b, mi31, and mi522 had no statistical differences between the QSG7701 and QGY7703 cell lines(Fig. 3).

### Correlation analysis

There was a significant correlation between mir3660 and mir9-3, mir31, and mir-4784 (Spearman correlation). There was a positive correlation between mir10b and mir31 and clinical stages. Sex was correlated with mir-6883. Age was not correlated with these genes.

### Classification model

**Figure 4:**
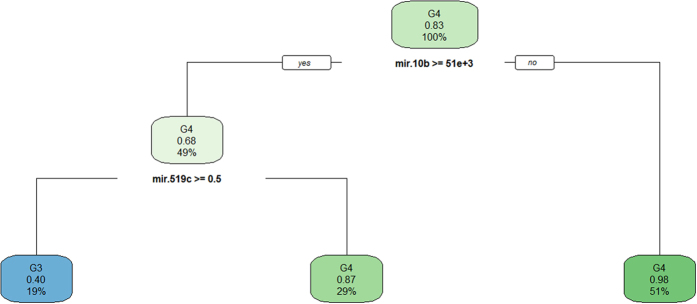
Decision tree model classified G3 and G4.

**Figure 5:**
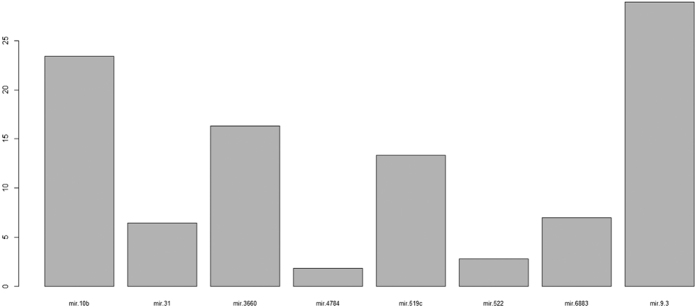
Adaboost model classified G3 and G4. The importance of gene sequencing.

**Figure 6:**
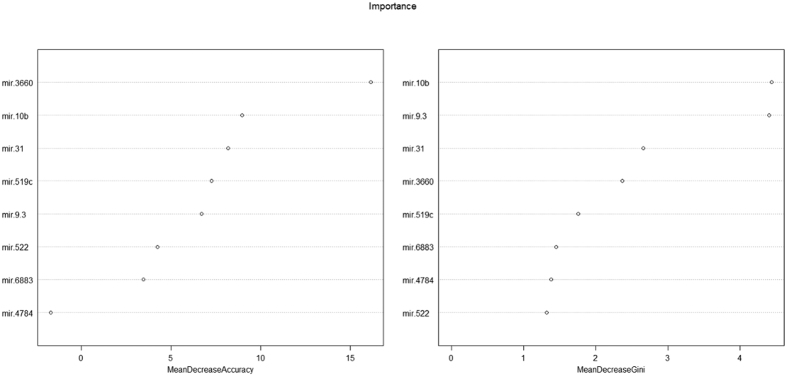
Random forests model classified G3 and G4. The importance of gene sequencing.

The decision tree classified all the data with misjudgment rate of 0.128 (Fig. 4). The decision tree model average misjudgment rate of 10-fold cross-validation was 0.25. While the AdaBoost classified all the data with misjudgment rate of 0 (Fig. 5), the AdaBoost model average misjudgment rate of 10-fold cross-validation was 0.158. The random forests classified all the data with misjudgment rate of 0 (Fig. 6). The random forests model average misjudgment rate of 10-fold cross-validation was 0.109.

## Discussion

Many studies have shown that microRNAs are involved in modulating gene expression, in HBV and HCV replication, and play an important role in the interaction between host and virus. Many studies have reported abnormal miRNA expression levels in liver tumors compared to normal tissue^16^,^17^,^18^. MicroRNAs are highly conserved, small, non-coding RNAs. Their average length is 22 nucleotides, and they have been shown to be main regulators of gene expression and to be correlated with many tumors, including HCC^9^. The occurrence and development of many diseases might be due to alterations in miRNA biogenesis^19^,^20^. The deregulation of miRNAs might affect oncogenes and tumor suppressor genes in human carcinogenesis^21^. Hung *et al*. reported that serum levels of miR-122 and let-7b were significantly lower in patients with cirrhosis or dysplastic nodules than in patients with HBV-HCC, indicating that they could be useful markers for the early diagnosis of HCC^22^. However, we found no significant difference in miR-122 and let-7b levels in our analysis of the 93 cases of HBV-HCC (G1) and 49 cases of adjacent normal controls (G2) groups. This difference might be due to different tissues that were analyzed.

Worldwide, persistent infection with HBV or HCV is the main cause of liver cirrhosis and HCC^23^. HBV-HCC is especially common in Asia. The TCGA is known for its goal of generating comprehensive, multi-dimensional maps of the key genomic changes in various types of cancers. In this study, we used the TCGA database to mine microRNAs involved in HBV-HCC that could be used for clinical diagnosis, treatment, and predicting survival. These microRNAs would also be a component of a precise medical approach. Although there have been many studies that used the TCGA database to mine genes involved in tumors, our study contains several innovations, and its results could guide future studies. We believe that the different etiologies of liver cancer are characterized by differentially expressed genes. Therefore, we only screened the same etiology, HBV, of liver cancer for DE genes, which was different from the approach of previous studies^15^. We performed a Kaplan–Meier survival analysis of the DE group and identified eight genes that had statistical significance. We set up three kinds of classification models, and compared their average 10-fold cross-validation misjudgment rates. Our classification model was able to predict the prognosis of patients with high accuracy.

We performed a survival analysis of the DE genes. The Kaplan–Meier survival analysis showed that eight genes (mir9-3, mir10b, mir31, mir519c, mir522, mir3660, mir4784, and mir6883) were statistically different. The eight genes had differences not only in expression but also in survival. We compared QSG7701, a human normal liver cell line, with three additional liver cancer cell lines (QGY7703, HepG2, and SMMC7721) using qPCR to test 8 microRNAs. The results show that the 8 microRNAs have differentiable high expression levels in the liver cancer cell lines (Fig. 3). Chen *et al*. reported that miR-9 was overexpressed in lung cancer tissues compared with peripheral normal tissues^24^, similar to our results. Studies^25^,^26^ have shown that miR-10b is a prognostic biomarker for melanoma and esophageal squamous cell carcinoma, similar to our conclusion. However, miR-31 expression correlated with a better outcome in advanced stage serous ovarian tumors^27^. This study by Creighton utilized only advanced stage cancers^27^; it did not also use early stage cancers. The expression levels of miR-31 were significantly increased in the patients with a high risk of recurrence compared to the low-risk patients, and higher expression of miR-31 correlated with a poor survival^28^. Our article supported Mitamura’s point of view^28^. Zhang reported miR-522 was upregulated in HCC cells; the overexpression miR522 could promote cell proliferation^29^. These previous studies support our findings. However, miR-519c and tumor status exhibited an inverse association in patient samples; miR-519c expression is closely associated with tumor progression in human cancer tissues^30^. It was different from our sample^30^; perhaps this was one of the reasons. The Cox proportional hazards regression model showed that mir10b, mir519c, mir3660, and mir6883 had statistically significant differences.

We performed a Spearman rank correlation analysis of the microRNAs (mir9-3, mir10b, mir31, mir519c, mir522, mir3660, mir4784, and mir6883) and clinical parameters (age, sex, and clinical stages). The results showed that there was an association between mir3660 and mir9-3, mir31, and mir-4784. The eight genes were not related to age. The results showed that mir10b and mir31 had positive a correlation with the clinical stage, which further indicated that mir10b and mir31 were associated with tumor progression. It is interesting to note that mir6883 was associated with sex; we speculated that mir6883 might be associated with sex hormones.

We included eight genes (mir9-3, mir10b, mir31, mir519c, mir522, mir3660, mir4784, and mir6883) in the ROC analysis to distinguish between G3 and G4 liver cancers. The results showed that three genes were statistically different, mir9-3, mir10b, and mir519c, with AUC values of 0.769, 0.738, and 0.676, respectively (p < 0.05). This suggested that the expression levels of these three genes can effectively distinguish between G3 and G4 liver cancers. However, the specificity and sensitivity were poor. Thus, we aim to establish a mathematical model to better distinguish between G3 and G4.

We used the binary classification of variables in three kinds of models, decision tree, AdaBoost, and random forests, to distinguish between G3 and G4. The 10-fold cross-validation suggested that the random forests model, with the smallest average misjudgment rate of 0.109, could effectively predict HBV-HCC survival. Mir9-3, mir10b, mir31, and mir3660 were very important to predict survival (Fig. 6). In practical application, however, the different gene sequencing platforms may result in errors. Nonetheless, based on our modeling experience, we believe that with proper control of the errors and precise sequencing, it is possible to establish a highly accuracy mathematical model to predict survival. We believed that with the reduction in cost of sequencing, we can use sequencing data to predict the prognosis of cancer patients.

In our genes validation tests, the selection of patients with plasma was inappropriate. Choosing a large number of liver cancer and normal adjacent tissue samples for validation of these miRNAs was the best choice. Due to some limitations, we selected the liver cancer and normal liver cell lines to verify these miRNAs, which could also provide meaningful information. Our discrepancy expression genes exhibit a high true-positive rate. The criteria to define differences in the screened genes were a 3-fold change for upregulation and 0.33-fold change for downregulation, with p < 0.05, which had a high standard. More importantly, we conducted a survival analysis with p < 0.05. Compared with the normal liver cell line, hepatocellular carcinoma cell lines had high expression of 8 microRNAs, albeit at different levels. We speculated the eight genes (mir9-3, mir10b, mir31, mir519c, mir522, mir3660, mir4784 and mir6883) might be related to HBV-HCC. Many studies have reported that the relationship between miRNAs and cancer. MiR-9 was overexpressed in lung cancer tissues^24^. MiR-9 acts as a poor prognostic biomarker in lung cancer and papillary thyroid cancer^31^,^32^. Studies^25^,^26^ have shown that miR-10b is a prognostic biomarker for melanoma and esophageal carcinoma. Higher expression of miR-31 correlated with poor survival^28^. MiR-522 was upregulated in HCC cells, and the overexpression of miR522 could promote cell proliferation^29^. These reports support our results. In addition, the mir9-3, mir10b, mir31, and mir522 of high-throughput sequencing data were high. The results were stable and reliable. Therefore, these genes (mir9-3, mir10b, mir31, and mir522) had a high true-positive rate. Four genes (mir519c, mir3660, mir4784 and mir6883) of high-throughput sequencing data were low, with many reads being 0. However, high-throughput sequencing can unavoidably yield errors. Therefore, there might be false positives in the four genes (mir519c, mir3660, mir4784 and mir6883). However, we cannot ignore the differences between cell lines and human tissues. In the future, these genes needed to be validated in human samples. Nevertheless, we believe that these eight genes have a high true-positive rate, and are beneficial to the diagnosis and treatment of liver cancer.

In conclusion, eight genes (mir9-3, mir10b, mir31, mir519c, mir522, mir3660, mir4784, and mir6883) were identified could predict survival in patients with HBV-HCC. There was a significant correlation between mir10b and mir31 and clinical stages (Spearman correlation, p < 0.05). Compared with the normal liver cell line, the 8 microRNAs have differentiable high expression levels in the liver cancer cell lines. A random forest model with average misjudgment rate 0.109 could effectively estimate the patient survival prognosis.

## Materials and Methods

### Patients and data

Liver cancer patients with risk factor for HBV infection (i.e., HBV-HCC miRNAseq and clinical data) were downloaded from the TCGA database on April 28, 2016. We used the python scripts and the Linux operating system to process the data. Cases of hepatitis C virus (HCV)-infected liver cancer and patients with missing data were excluded. There were G1 and G2 included in the study. Table 2 shows the characteristics of the G1 and G2.

### Gene screening

We performed differential expression gene screenings. We compared 93 liver cancer cases with 49 normal controls. We calculated the fold changes between the genes using the edgeR package (http://www.bioconductor.org/packages/release/bioc/html/edgeR.html) in the R language. We also used F-tests for quasi-likelihood assessments. The criteria to define differences in screened genes were a threefold change for upregulation and a 0.33-fold change for downregulation, with p < 0.05.

### Heat maps

Two heat maps were constructed (93 liver cancer cases vs. 49 normal controls). The darkness of the heat map color represents the gene expression level: the darker the color, the higher the level of gene expression. We used the gplots package to draw the heat maps (https://cran.r-project.org/web/packages/gplots/).

### Statistical analysis

We applied the R 3.2.2 (https://www.r-project.org/) language for data processing. We estimated the dispersion for each gene to look for differentially expressed genes according to the median value of gene expression divided into high and low expression groups. In the Kaplan–Meier survival analysis, we used the log-rank testing method for the univariate analysis. We used principal component analysis for the selected microRNAs in 78 patients (G3 and G4). And we compared QSG7701, a human normal liver cell line, with three liver cancer cell lines (QGY7703, HepG2, and SMMC7721) using qPCR to test 8 microRNAs. We used reverse transcription of 8 microRNAs. We then used the primers synthesized by RiboBio Company for the qPCR. We used a Cox proportional hazards regression model for multivariate analysis. We excluded patients with no follow-up time. Finally, 92 patients with detailed follow-up times were included in the study. We performed a ROC analysis for genes that were statistically significant in the survival analysis. Thirteen cases of short survival time and 65 cases of long survival time for liver cancer were included into the ROC analysis, for which SPSS22.0 statistical software was used. We performed a correlation analysis between the microRNAs (mir9-3, mir10b, mir31, mir519c, mir522, mir3660, mir4784, and mir6883) and clinical parameters (age, sex, and clinical stages) and used the Spearman correlation coefficient. A p-value less than 0.05 was considered statistically significant.

### Classification model

The data from 78 patients (mir9-3, mir10b, mir31, mir519c, mir522, mir3660, mir4784, and mir6883) were used to establish classification models (decision tree, AdaBoost, and random forests) to better distinguish G3 and G4 of liver cancers. We used the ‘rpart’ packet (https://cran.r-project.org/web/packages/rpart/) to establish the decision tree model. We also used the ‘adabag’ package (https://cran.r-project.org/web/packages/adabag/index.html) to establish the bagging model. And we used the ‘randomForests’ packet (https://cran.r-project.org/web/packages/randomForest/) to establish the random forest model. We compared the three kinds of classification models by the average misjudgment rates of their 10-fold cross-validations.

## Additional Information

**How to cite this article:** Zhen, Y. *et al*. Several microRNAs could predict survival in patients with hepatitis B-related liver cancer. *Sci. Rep.* **7**, 45195; doi: 10.1038/srep45195 (2017).

**Publisher's note:** Springer Nature remains neutral with regard to jurisdictional claims in published maps and institutional affiliations.

## Supplementary Material

Supplementary Information

## Figures and Tables

**Table 1:** Cox proportional hazards regression model for multivariate analysis.

Items	Classification	P
mir9-3	high expression vs lower expression	>0.05
mir10b	high expression vs lower expression	<0.05
mir31	high expression vs lower expression	>0.05
mir519c	high expression vs lower expression	<0.05
mir522	high expression vs lower expression	>0.05
mir3660	high expression vs lower expression	<0.05
mir4784	high expression vs lower expression	>0.05
mir6883	high expression vs lower expression	<0.05
age	<= 53 vs > 53	>0.05
gender	male vs female	>0.05
T-stage	I + II vs III + IV	>0.05
tumor grade	I + II vs III + IV	>0.05

**Table 2:** Characteristics of HBV-HCC patients vs normal control.

Category	HBV-HCC (93)	normal control (49)	P
Age, Mean ± SD	54.3	61.6	<0.05
Gender			<0.05
Female	16	22	
Male	77	27	
Stage			<0.05
Stage I + II	83	29	
Stage III + IV	8	12	
AFP			>0.05
AFP < 20	44	15	
AFP > 20	46	18	
Tumor status			<0.05
Tumor free	66	21	
With tumor	26	20	
History other malignancy			>0.05
Yes	5	4	
No	88	45	
