# Supplementary material for: Several microRNAs could predict survival in patients with hepatitis B-related liver cancer
Source: Sci Rep. 2017 Mar 21;7:45195. doi: 10.1038/srep45195 (PMC5359660; doi:10.1038/srep45195)
Supplement: Supplementary Information [file srep45195-s1.doc]

**Supplementary materials** **for** **“Several microRNAs could predict survival in patients with hepatitis B-related liver cancer”**

Ye Zhen, Zhao Xinghui, Wu Chao, Zhao Yi, Chen Jinwen, Gao Ruifang, Zhang Chao, Zhao Min, Guo Chunlei, Fang Yan, Du Lingfang, Shen Long, Shen Wenzhi, Luo Xiaohe, Xiang Rong


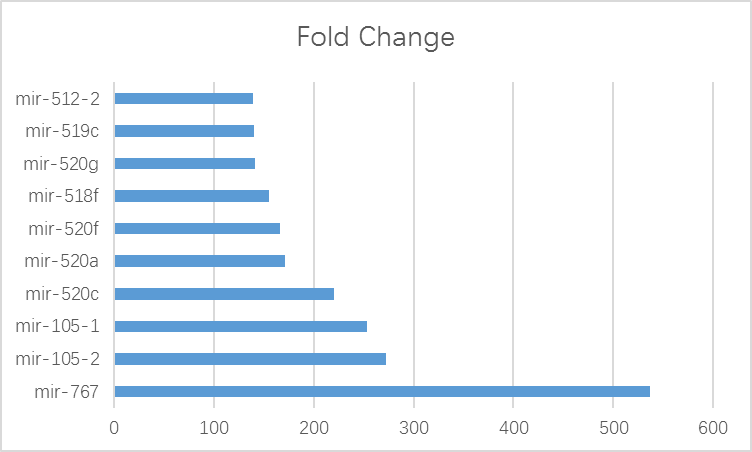


S-1. The top 10 up-regulated expression genes.


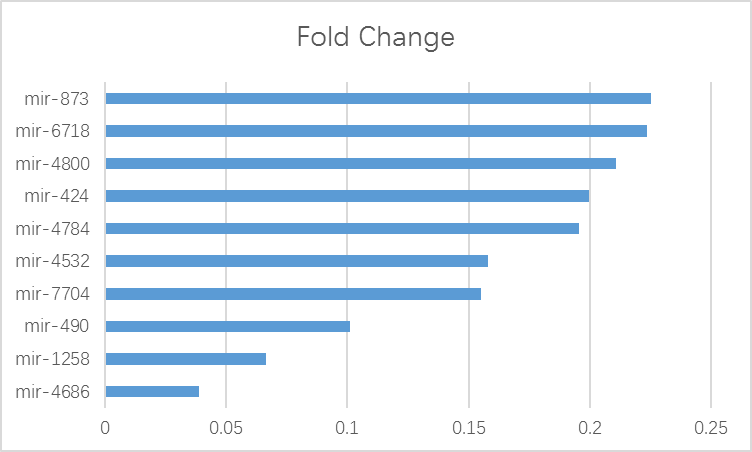


S-2. The top 10 downregulated expression genes.


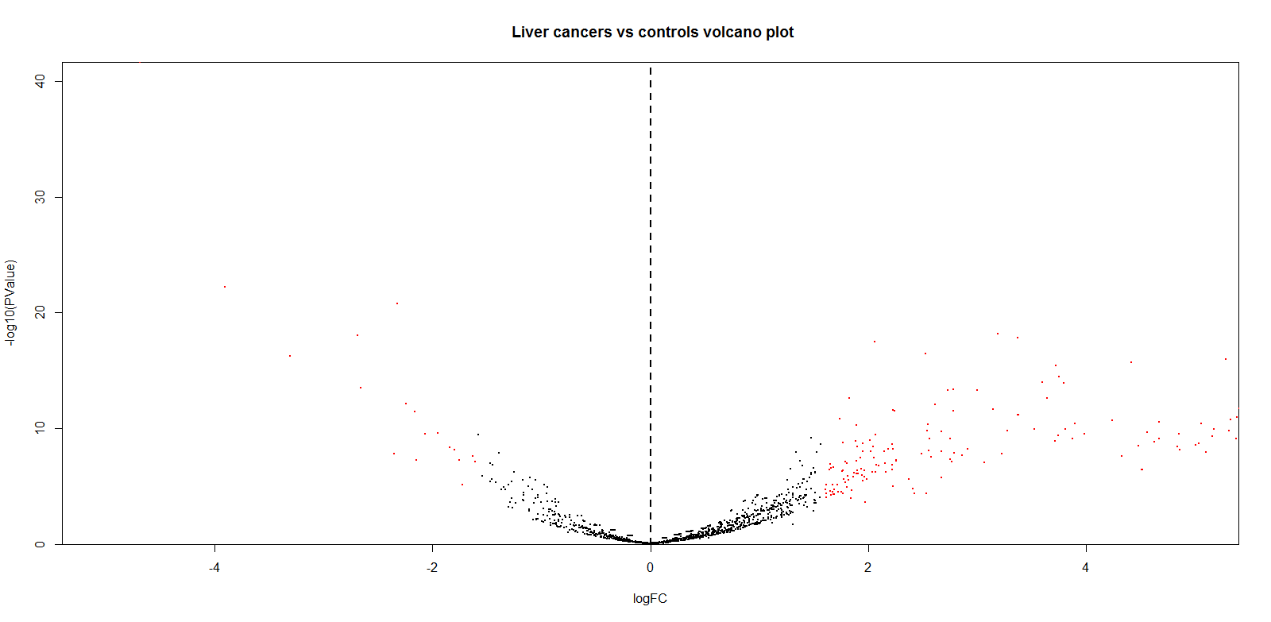


S-3. The red dots represent differentially expressing genes.


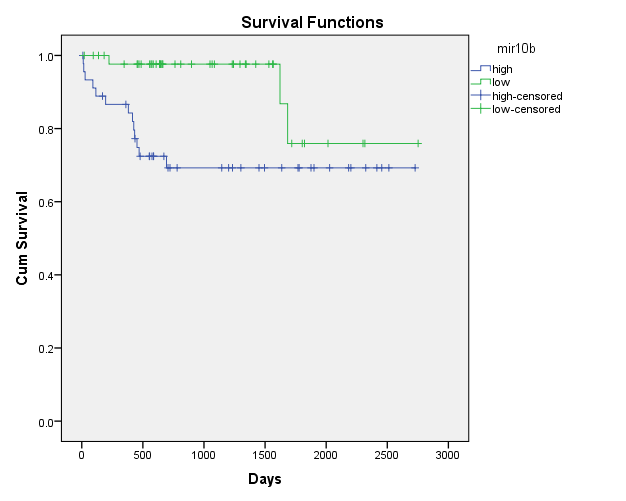


S-4. Kaplan-Meier survival analysis indicating that low expression of mir10b is superior to high expression, p < 0.05


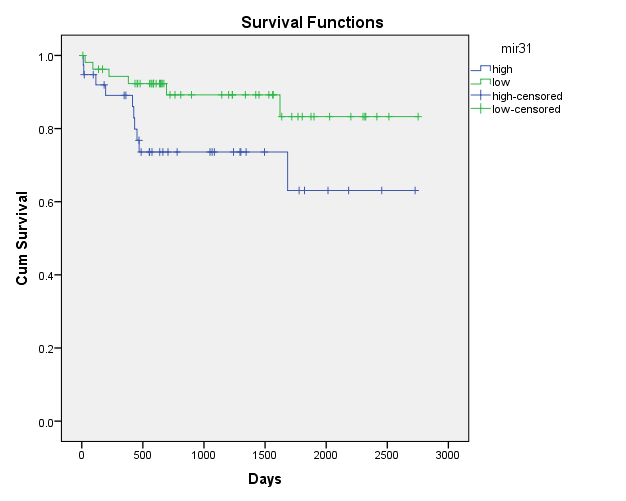


S-5. Kaplan-Meier survival analysis indicating that low expression of mir31 is superior to high expression, p < 0.05


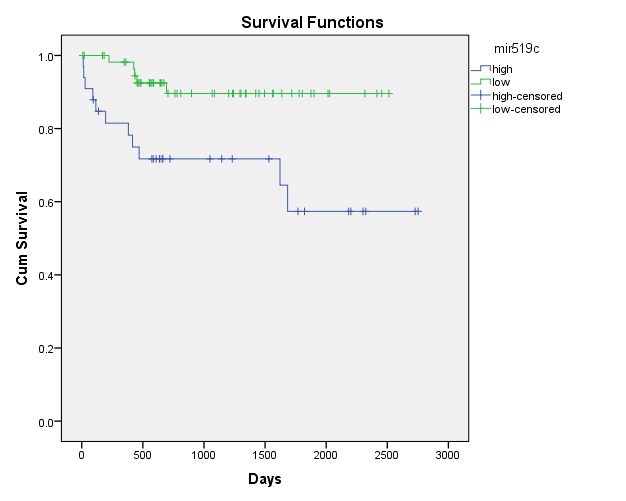


S-6. Kaplan-Meier survival analysis indicating that low expression of mir519c is superior to high expression, p < 0.05


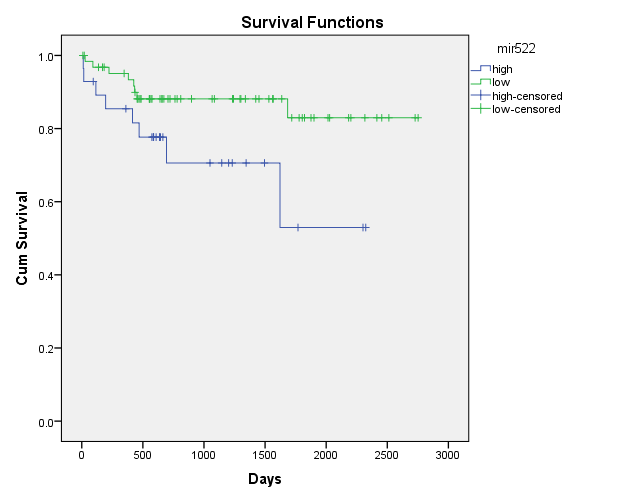


S-7. Kaplan-Meier survival analysis indicating that low expression of mir522 is superior to high expression, p < 0.05


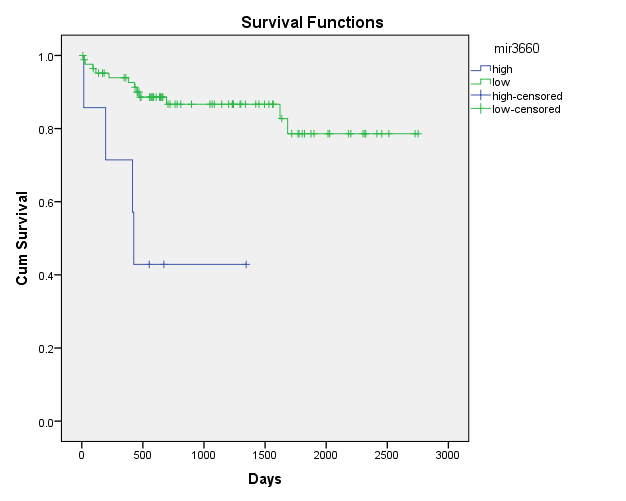


S-8. Kaplan-Meier survival analysis indicating that high expression of mir3660 is superior to low expression, p < 0.05


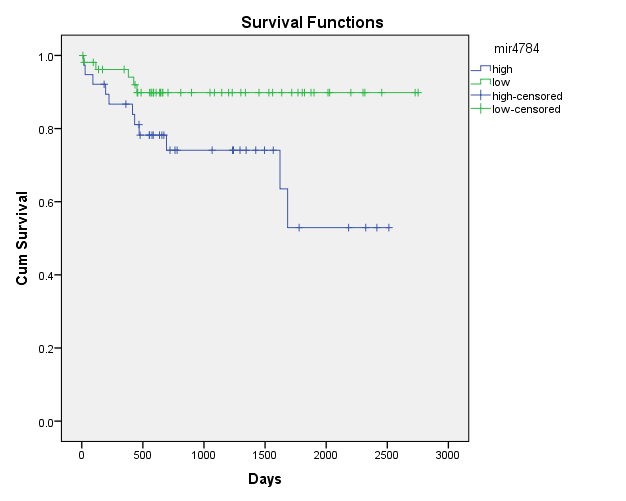


S-9. Kaplan-Meier survival analysis indicating that low expression of mir4784 is superior to high expression, p < 0.05


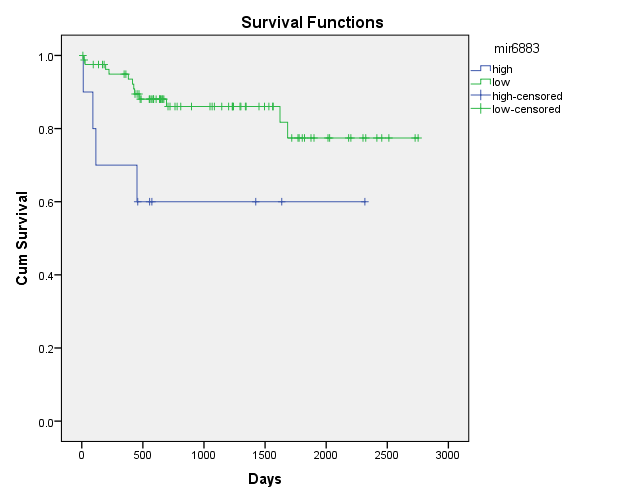


S-10. Kaplan-Meier survival analysis indicating that low expression of mir6883 is superior to high expression, p < 0.05


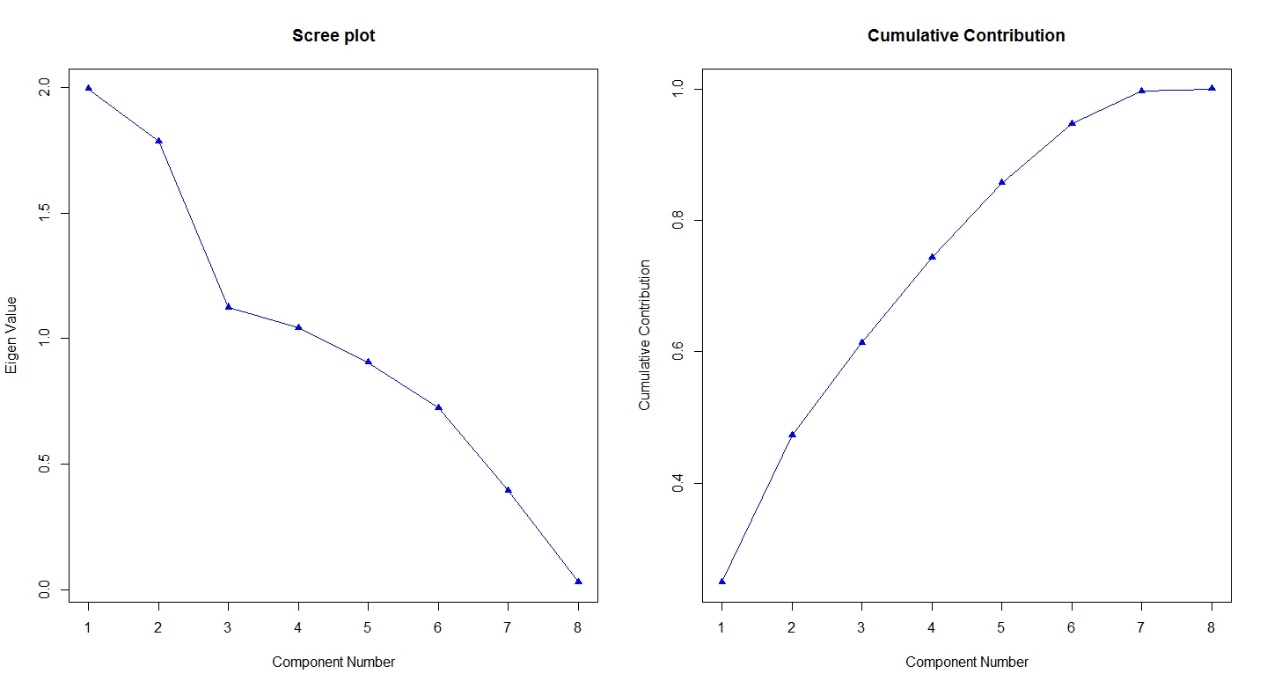


S-11. The scree plot and cumulative contribution of principal component analysis.

S-12. The characteristics of 78 cases of G3 and G4 patients’ data.
